# Transcriptional Profiling of Midgut Immunity Response and Degeneration in the Wandering Silkworm, *Bombyx mori*

**DOI:** 10.1371/journal.pone.0043769

**Published:** 2012-08-24

**Authors:** Qiuyun Xu, Anrui Lu, Guohua Xiao, Bing Yang, Jie Zhang, Xuquan Li, Jingmin Guan, Qimiao Shao, Brenda T. Beerntsen, Peng Zhang, Chengshu Wang, Erjun Ling

**Affiliations:** 1 Key Laboratory of Insect Developmental and Evolutionary Biology, Institute of Plant Physiology and Ecology, Shanghai Institutes for Biological Sciences, Chinese Academy of Sciences, Shanghai, People’s Republic of China; 2 Department of Veterinary Pathobiology, University of Missouri, Columbia, Missouri, United States of America; 3 National Laboratory of Plant Molecular Genetics, Institute of Plant Physiology and Ecology, Shanghai Institutes for Biological Sciences, Chinese Academy of Sciences, Shanghai, People’s Republic of China; Kyushu Institute of Technology, Japan

## Abstract

**Background:**

Lepidoptera insects have a novel development process comprising several metamorphic stages during their life cycle compared with vertebrate animals. Unlike most Lepidoptera insects that live on nectar during the adult stage, the *Bombyx mori* silkworm adults do not eat anything and die after egg-laying. In addition, the midguts of Lepidoptera insects produce antimicrobial proteins during the wandering stage when the larval tissues undergo numerous changes. The exact mechanisms responsible for these phenomena remain unclear.

**Principal Findings:**

We used the silkworm as a model and performed genome-wide transcriptional profiling of the midgut between the feeding stage and the wandering stage. Many genes concerned with metabolism, digestion, and ion and small molecule transportation were down-regulated during the wandering stage, indicating that the wandering stage midgut loses its normal functions. Microarray profiling, qRT-PCR and western blot proved the production of antimicrobial proteins (peptides) in the midgut during the wandering stage. Different genes of the immune deficiency (Imd) pathway were up-regulated during the wandering stage. However, some key genes belonging to the Toll pathway showed no change in their transcription levels. Unlike butterfly (*Pachliopta aristolochiae*), the midgut of silkworm moth has a layer of cells, indicating that the development of midgut since the wandering stage is not usual. Cell division in the midgut was observed only for a short time during the wandering stage. However, there was extensive cell apoptosis before pupation. The imbalance of cell division and apoptosis probably drives the continuous degeneration of the midgut in the silkworm since the wandering stage.

**Conclusions:**

This study provided an insight into the mechanism of the degeneration of the silkworm midgut and the production of innate immunity-related proteins during the wandering stage. The imbalance of cell division and apoptosis induces irreversible degeneration of the midgut. The Imd pathway probably regulates the production of antimicrobial peptides in the midgut during the wandering stage.

## Introduction

Insects such as *Drosophila melanogaster* live on rotten fruit and food containing many microbes, yet they still survive. The insect midgut provides innate immunity during the feeding stage against many pathogens ingested with their food. Under the delicate control of the midgut innate immune system, the pathogenic microbes can be specifically eliminated with minimal disruption to commensal and mutualistic bacteria [1], [2]. Thus, these insects have evolved an effective defense system, which has become a research focus. The insect gut is a continuous tube that starts from the mouth and ends at the anus. It is composed of three parts: the foregut, midgut, and hindgut. In insects, ingested food is stored and partially digested in the foregut, whereas the midgut is the primary site of digestion and absorption of nutrients. In the hindgut, some water and salts are absorbed to balance the hemolymph osmotic pressure during the process of feces formation [3].

All insects undergo metamorphosis, by which they develop from larvae into adults under the control of juvenile hormone (JH) and 20-hydroxyecdysone (20-E) [4], [5]. For insects undergoing incomplete metamorphosis, they have three stages: egg, nymph, and adult. Most insects show complete metamorphosis with obvious morphological changes between the egg, larva, pupa, and adult stages [6]. For each stage of complete metamorphosis, the internal tissues and organs undergo great change.

During metamorphosis, the insect midgut also undergoes many morphological changes. The midgut stem cells differentiate into a simple cuboidal epithelium, which separates the remnant epithelium from the basement membrane and releases it into the lumen as a yellow body [7], [8]. The yellow body then undergoes apoptosis and autophagy to re-utilize and absorb nutrients [7], [8]. Finally, the pupal epithelial cells differentiate and develop into the adult midgut. In *Drosophila*, the intestinal stem cells are located in the basal membrane and distribute evenly along the midgut [9], [10], [11]. Under the control of the Delta-Notch signaling pathway, these stem cells divide and differentiate to become the adult midgut, which performs the functions of food digestion and nutrient absorption during the adult stage [11], [12]. Interestingly, there are several layers (the larval midgut inside, transient pupal midgut in the middle, and the new emerged adult midgut outside) of pupal midgut observed in *Drosophila* [13]. In some insects, like *Tenebrio molitor*, many pouches appear in the adult midgut, which is very different from the larval midgut [14]. In *Bombyx mori*, one typical Lepidoptera insect, the midgut continues to degenerate after entering the wandering and pupal stages [15], [16]. After egg laying, silkworm moths do not eat anything and soon die. However, many Lepidoptera adults need functional midguts for nectar ingestion and digestion [17]. Interestingly, *Manduca sexta* produces a cocktail of potent antimicrobial proteins and peptides (AMPs), such as hemolin, lysozyme, and phenoloxidase, in the lumen of wandering stage larvae [18]. AMPs produced in the midguts of wandering stage larvae are believed to be involved in removing midgut bacteria before metamorphosis. However, little is known about the mechanism or its regulation.

Here, we performed a microarray assay of gene transcriptional changes in the midgut between the feeding stage (12 h on day 3 of fifth larval stage, V-3:12 h) and the wandering stage (3 h after the initiation of wandering, W:3 h) to determine why silkworm midguts continue to degenerate and if they produce AMPs during the wandering stage. Our results indicate many genes associated with metabolism, digestion, and ion and small molecule transportation are down-regulated during the wandering stage when ecdysteroid is high in the hemolymph. These changes may cause abnormal absorption in the midgut. In addition, cell division in the midgut ceases, but extensive cellular apoptosis was observed at the end of the wandering stage. Thus, the imbalance of cell division and apoptosis eventually drives the degeneration of the midgut. Microarray profiling, qRT-PCR, and Western blot proved the production of antimicrobial proteins/peptides during the wandering stage. Some key genes belonging to the Toll pathway showed no obvious changes, whereas genes of the immune deficiency (Imd) pathway were up-regulated. This suggests that the Imd pathway regulates the production of AMPs in the wandering stage midgut.

## Results and Discussion

### Transcriptional Changes between the Feeding Stage and Wandering Stage

**Figure 1:**
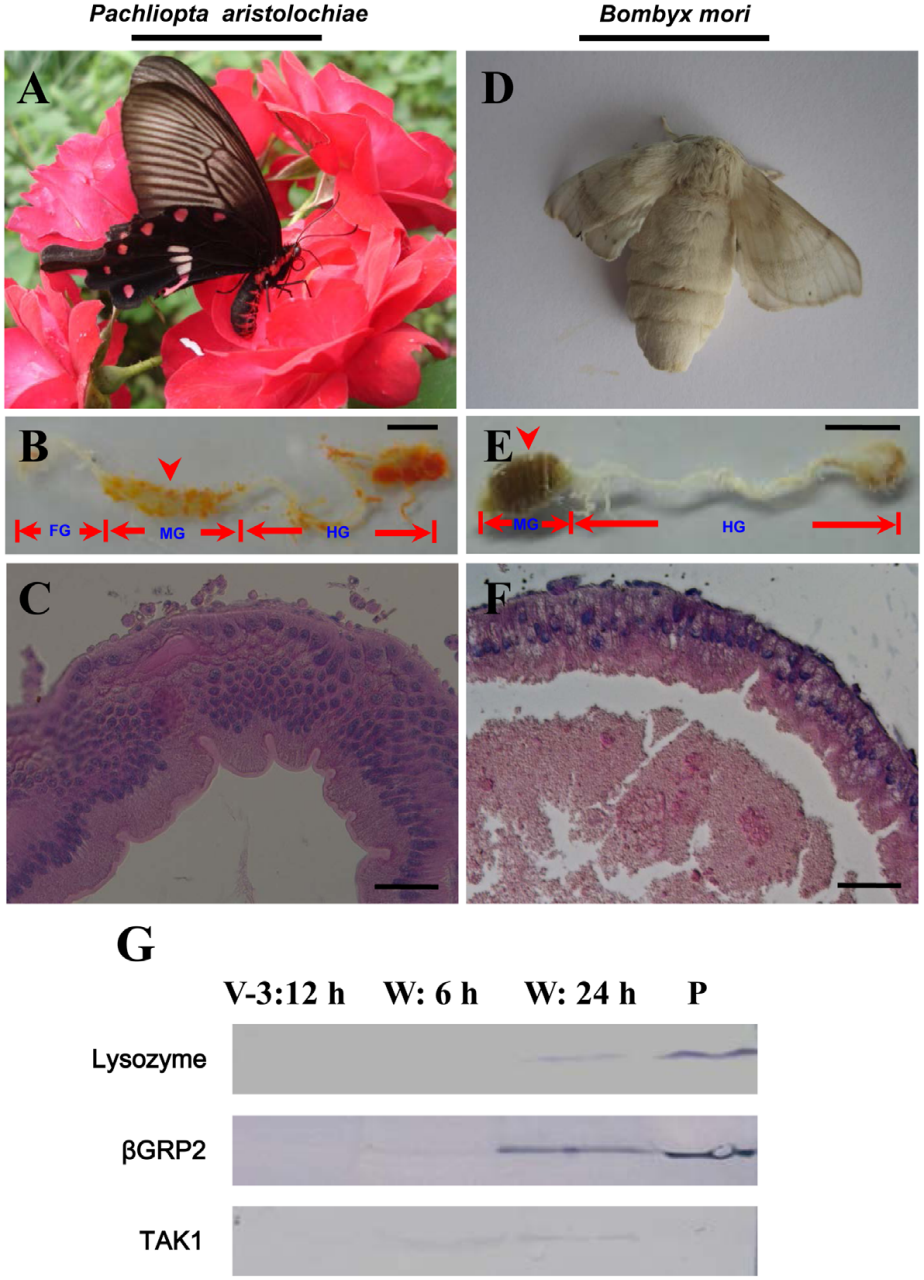
Different morphology of silkworm moth midgut compared with that of a butterfly. (A–C) Morphology of a butterfly (*Pachliopta aristolochiae*) and its adult midgut; (D–F) Morphology of a silkworm moth (*Bombyx mori*) and its adult midgut. The silkworm moth’s foregut (FG) is not easily dissected with the midgut. In (B and E), the arrow-indicated part of midgut (MG) was sampled for histological study as shown in (C and F). The butterfly (A) ingests nectar and the midgut has many layers of cells (C) and appears in good condition. However, the midgut of silkworm moth is full of yellow body debris that cannot be excreted (E) and appears in weak condition due to one layer of cells (F). The silkworm moth (D) does not ingest anything and dies after egg-laying. FG: foregut; MG: midgut; HG: hindgut. (G) Three immunity-related proteins were significant expressed in the midgut during the wandering stage. Some proteins, such as lysozyme, βGRP2 (antibody against *M. sexta* βGRP2; 31% similarity to *B. mori* βGRP2), and TAK1 (antibody against mouse TAK1; 70% similarity to *B. mori* TAK1) were detected in the midgut during the wandering stage. Plasma (P) was from larvae injected with *E. coli*. For each lane, approximately 10 µg cell lysate was loaded. Bars: (B and E) 4 mm; (C and F): 50 µm.

**Figure 2:**
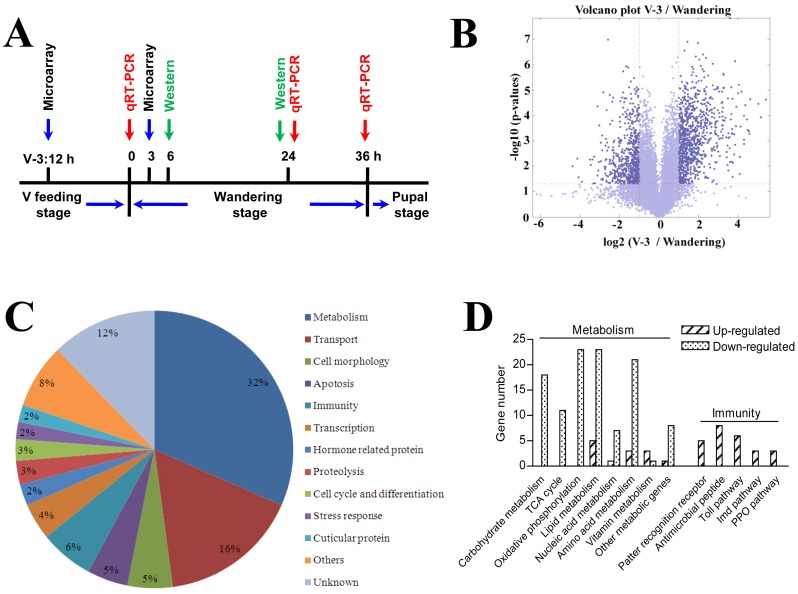
General statistics on the genes regulated between the feeding stage (V-3:12 h) and wandering stage (W:3 h). (A) The times of different sampling. We selected larvae at 12 h on day 3 of the fifth larval stage (V-3:12 h), or 3 h after the initiation of the wandering stage (W: 3 h), which were dissected for the microarray. The time points for Western blot and qRT-PCR were determined according to the preliminary work with lysozyme. (B) Volcano plots depicting estimated fold change (log2, X-axis) and statistical significance (−log10 P value, Y-axis). Each point represents a gene, and colors correspond to the range of negative log10 P and log2 fold-change values. (C) GO categories of differentially transcribed genes between the feeding and wandering stages. (D) The numbers of up- and down-regulated genes associated with various metabolic events and innate immunity.

Butterfly and silkworm belong to Lepidoptera insects. The adult butterfly (*Pachliopta aristolochiae*) living on nectar (Fig. 1A), has a midgut with many layers of cells (Fig. 1B and 1C) for nectar ingestion and digestion [17]. The butterfly midgut is covered by many unknown tissues that contain yellow pigments. No midgut content was visible in the midgut (Fig. 1C) likely because the fluid nectar was easily lost during the process of dissection. The silkworm moth midgut is composed of one layer of cells in most regions of the midgut and is still full of yellow bodies (Fig. 1E and 1F), and the midgut is not suitable food digestion. Obviously, the midgut of silkworm moth has a poor progress of development since the wandering stage (Fig. 1C and 1F). Beside morphological changes in the midguts of Lepidoptera insects, many immunity-related proteins are produced during the wandering stage in *M. sexta* [18], [19]. Several immunity related proteins were specifically detected in the midguts of silkworm during the wandering stages (Fig. 1G). For example, lysozyme and βGRP2 had the highest protein levels in the midgut at 24 h after the initiation of wandering stage (W:24 h). TAK1, a very important component in the Imd pathway [20], [21], was obvious expressed at 6 h after the initiation of wandering stage (W:6 h). Obviously, just like *M. sexta*, there are also many immunity related proteins expressed in the silkworm midgut during the wandering stage. To date, there has been no explanation why the midguts of silkworms degenerate or whether silkworms produce antimicrobial proteins in their midguts after entering the wandering stage. For these reasons, we did a microarray to analyze gene transcription between the feeding stage and wandering stage (Fig. 2A). The results show a volcano plot that indicates that the transcript levels of many genes changed dramatically (Fig. 2B). Totally there are 399 genes that were differentially regulated. Among them, 155 genes (39%) were up-regulated and all others were down-regulated during the wandering stage (Table S1). These genes were classified into 13 families according to GO analysis (Fig. 2C). Metabolism and transport genes were the two largest families, with 126 (32%) and 65 (16%) genes whose transcription levels changed. Other categories included genes concerned with immunity (6%), cell morphology (5%), apoptosis (5%), and transcription (4%). In addition, there were 49 genes of unknown functions, among which 21 genes were down-regulated and 28 were up-regulated.

### Metabolism

Thirty-two percent of the differentially regulated genes were associated with metabolism, such as the tricarboxylic acid cycle (TCA cycle), oxidative phosphorylation, carbohydrate metabolism, lipid metabolism, nucleic acid metabolism, amino acid metabolism, and vitamin metabolism (Fig. 2D). Most of those genes (113 genes) were down-regulated after entering the wandering stage, particularly those genes associated with carbohydrate metabolism, the TCA cycle, and oxidative phosphorylation (Fig. 2D). The results indicate that many metabolic activities of the midgut were probably adversely affected during the wandering stage by the down-regulation of transcription of these genes.

### Hormones

**Figure 3:**
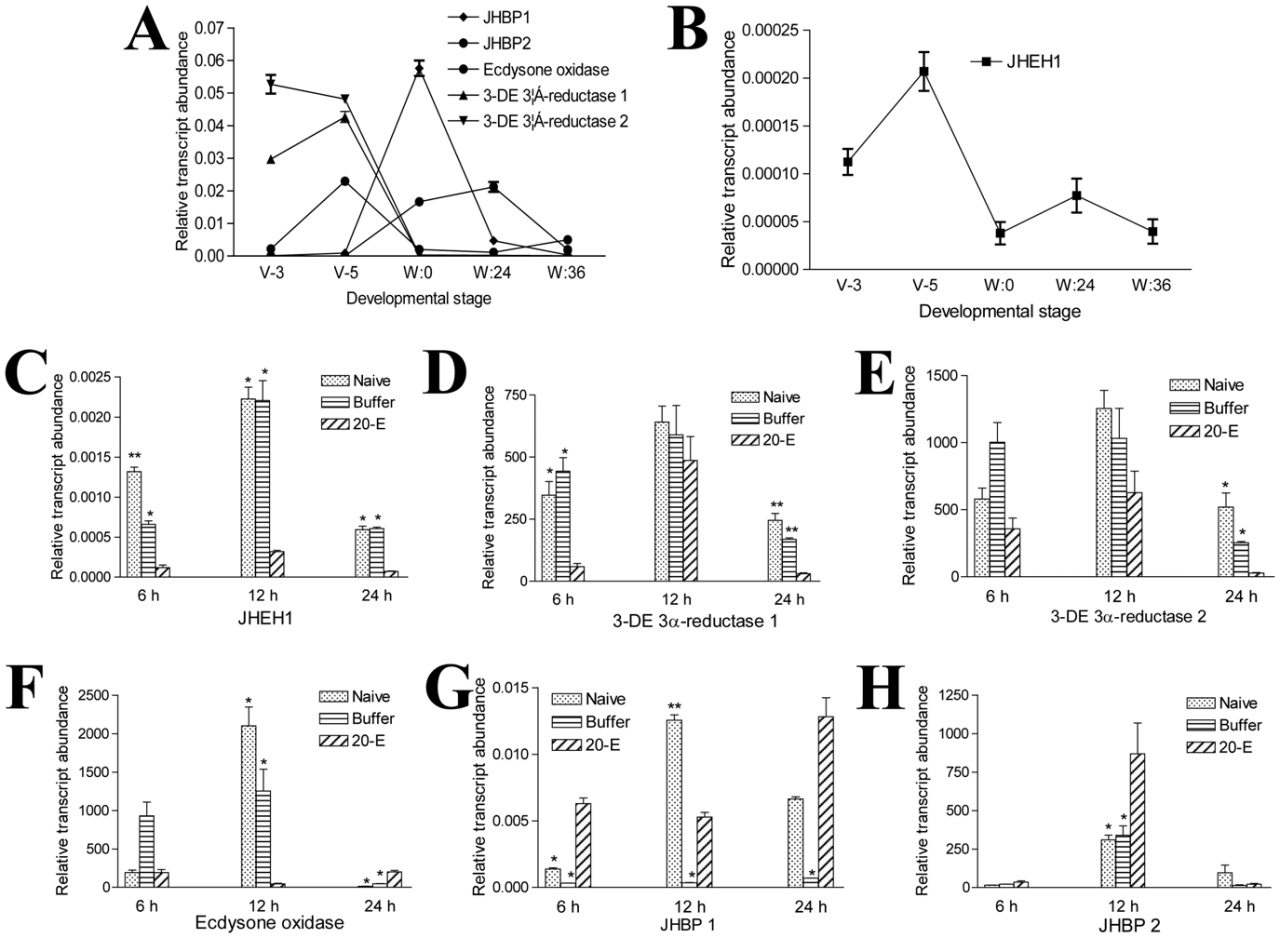
Genes concerned with regulation of hormones were differentially transcribed in the midgut. (A, B) Transcriptional changes of two JHBP genes, two 3-DE 3α-reductase genes, ecdysone oxidase and JHEH1 were different during development. The two JHBP genes and ecdysone oxidase were up-regulated, but the two 3-DE 3α-reductase genes and JHEH1 were down-regulated in the midgut during the wandering stages. (C–H) Influence of 20-E injection on the transcription of the above genes. JHEH1 and 3-DE 3α-reductase were down-regulated in the midgut when the larvae were injected with 20-E. However, the remaining genes responded to 20-E inconsistently. *p<0.05; **p<0.001.

Insect development differs from advanced vertebrate animals by the three or four rounds of metamorphosis that occur during their life cycle. These metamorphic events are coordinately controlled by JH and 20-E [4], [5]. The microarray assay showed that the transcript levels of several genes associated with hormone degradation were reduced during the wandering stage (Table S1). For example, genes encoding 3-dehydroecdysone 3α-reductase (3-DE 3α-reductase 1 and 3-DE 3α-reductase 2) and ecdysone oxidase, which are responsible for ecdysteroid degradation, changed considerably during development [22] (Fig. 3A). Another gene encoding juvenile hormone epoxide hydrolase-like protein 1 (JHEH1), which can convert JH into juvenile hormone diol [23], was also down-regulated (Fig. 3B). JH binding protein (JHBP) can bind with JH to form a JH-JHBP complex [24]. When there is no juvenile hormone esterase (JHE), JHBP can protect JH by forming a complex with it. However, if JHE is present, JHBP will help JHE to specifically detect the complex for subsequent JH hydrolysis [25]. Two JHBP genes were up-regulated on the wandering stage (Fig. 3A). When larvae on V-3 were injected with 20-E, JHEH1, 3-DE 3α-reductase 1, and 3-DE 3α-reductase 2 were all down-regulated at 24 h as compared to the naïve and buffer injection (Fig. 3C–3E). However, ecdysone oxidase and JHBP1 were down-regulated at 12 h, but up-regulated at 24 h post-injection (Fig. 3F and 3G). JHBP2 was up-regulated at 12 h as compared to the naïve and buffer injection (Fig. 3H).

**Figure 4:**
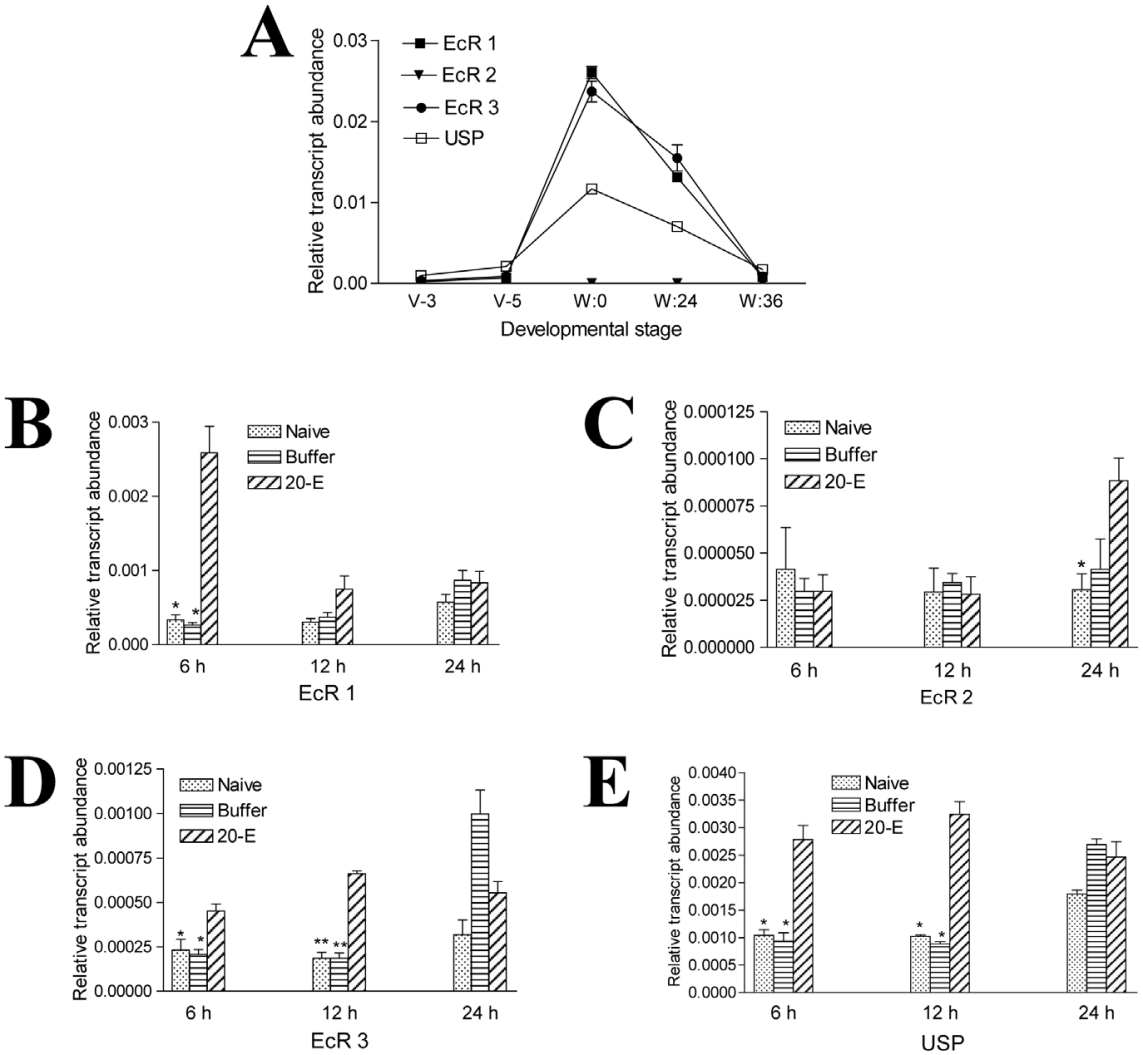
Ecdysone receptors (EcR) and ultraspiracle (USP) proteins are under the control of 20-E. (A) EcR and USP genes were quickly up-regulated when entering the wandering stage. The transcription levels of these genes were low during the feeding stage and the end of the wandering stage. (B–E) 20-E injection induced the transcription of EcR1, EcR3, and USP during the first 12 h compared with naive or buffer injection. EcR2 was up-regulated at 24 h after 20-E injection. *p<0.05; **p<0.001.

The EcR-USP (EcR: ecdysone receptor; USP: ultraspiracle protein) complex responds to the change of 20-E to initiate metamorphosis in insects [26]. In *Tribolium castaneum*, EcR and USP mediate midgut remodeling through a 20-E signal [27]. During the wandering stage, the transcription of three EcRs increased to their maximum, and then decreased within 36 h to almost the same level as during the feeding stage (Fig. 4A). The levels of the USP transcript showed a similar pattern (Fig. 4A). The changes in transcript levels of EcR and USP upon 20-E injection also showed a similar change tendency. They increased at different time (Fig. 4B–4E). This indicates that the increasing level of ecdysteroid in the hemolymph also regulates EcR and USP transcription in the midgut.

### Heat Shock Proteins

**Figure 5:**
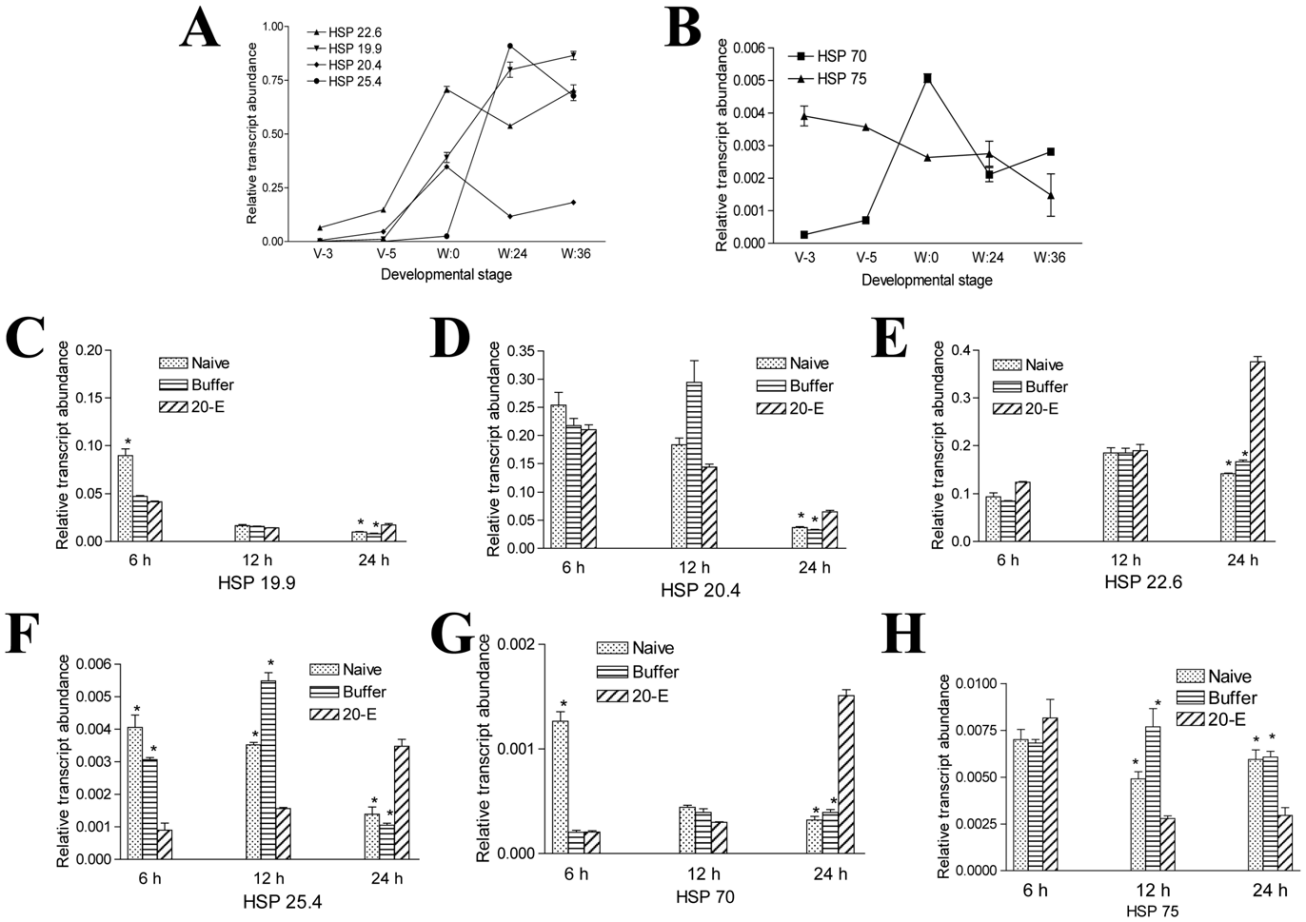
Regulation of HSPs in the midgut during the wandering stage. (A, B) The transcription levels of HSP 22.6, HSP 19.9, HSP 20.4, HSP 25.4, and HSP 70 were up-regulated in the midgut during development. Only HSP 75 was down-regulated. (C–H) Effect of 20-E on HSPs. HSP 19.9, HSP 20.4, HSP 22.6, HSP 25.4, and HSP 70 were up-regulated at 24 h after 20-E injection as compared to the naïve and buffer injection. HSP 75 was down-regulated after 20-E injection. *p<0.05.

The transcriptions of several heat shock proteins (HSPs) were up-regulated in the midgut during the wandering stage (Table S1). The qRT-PCR showed that the transcript levels of HSP 22.6, HSP 19.9, HSP 20.4, HSP 25.4, and HSP 70 were up-regulated in the midgut during development (Fig. 5A and 5B). HSP 75 is different; it had higher transcript levels during the feeding stage than during the wandering stage (Fig. 5B). When larvae (V-3) were injected with 20-E, the transcription of HSP 19.9, HSP 20.4, HSP 22.6, HSP 25.4, and HSP 70 significantly increased at 24 h post-injection (Fig. 5C–5G). However, 20-E negatively regulates HSP 75 transcription at 12 and 24 h as compared to the naïve and buffer injection (Fig. 5H).

HSPs are involved in protecting animals under various stresses and thermal injury and are involved in development [28], [29]. HSPs are divided into five groups according to their molecular weights, HSP 100, HSP 90, HSP 70, HSP 60, and small HSP (sHSP) [30]. The molecular weights of sHSPs vary from 12 kDa to 42 kDa [30], [31]. sHSPs connect with the cell nuclei, cytoskeleton, and membrane, and can also bind to denatured proteins as chaperonins, preventing irreversible coagulation under stress conditions [32]. In the wandering stage, many metabolism- and transport-related genes were down-regulated (Table S1). This may cause a stress in the midgut for normal physiological functions. The up-regulated transcription of sHSPs (HSP 19.9, HSP 20.4, HSP 22.6 and HSP 25.4) could represent a response by the midgut in an effort to maintain its integrity under this stress.

### Proteases

Serine proteases (SPs) and serine protease homologs (SPHs) have many physiological functions, such as innate immunity, development, digestion, and signal transduction [33], [34]. In the silkworm genome, there are 51 SP and 92 SPH genes [35]. The transcription of four SP genes (serine protease 54, trypsinogen-like protein, 35 kD protease and trypsin) and three SPH genes (30 kD protease A precursor, trypsin and trypsinogen-like protein) were down-regulated after entering the wandering stage (Table S1). In addition, 10 genes associated with protein digestion were down-regulated in the wandering stage (Table S1). One gene for protein proteolysis was up-regulated. However, in the prepupal midgut of *Heliothis virescens*, there are many hydrolytic enzymes [8]. In *M. sexta*, SPH1 and SPH2 have a very similar amino acid sequence to SP, but have no enzyme activity because of the loss of one or more catalytic residues [36], [37]. However, SPH1 and SPH2 have innate immunity functions: *M. sexta* SPHs are necessary for prophenoloxidase (PPO) activation [34]. The exact physiological functions of SP and SPH in the midgut deserve further study.

### Immunity

**Figure 6:**
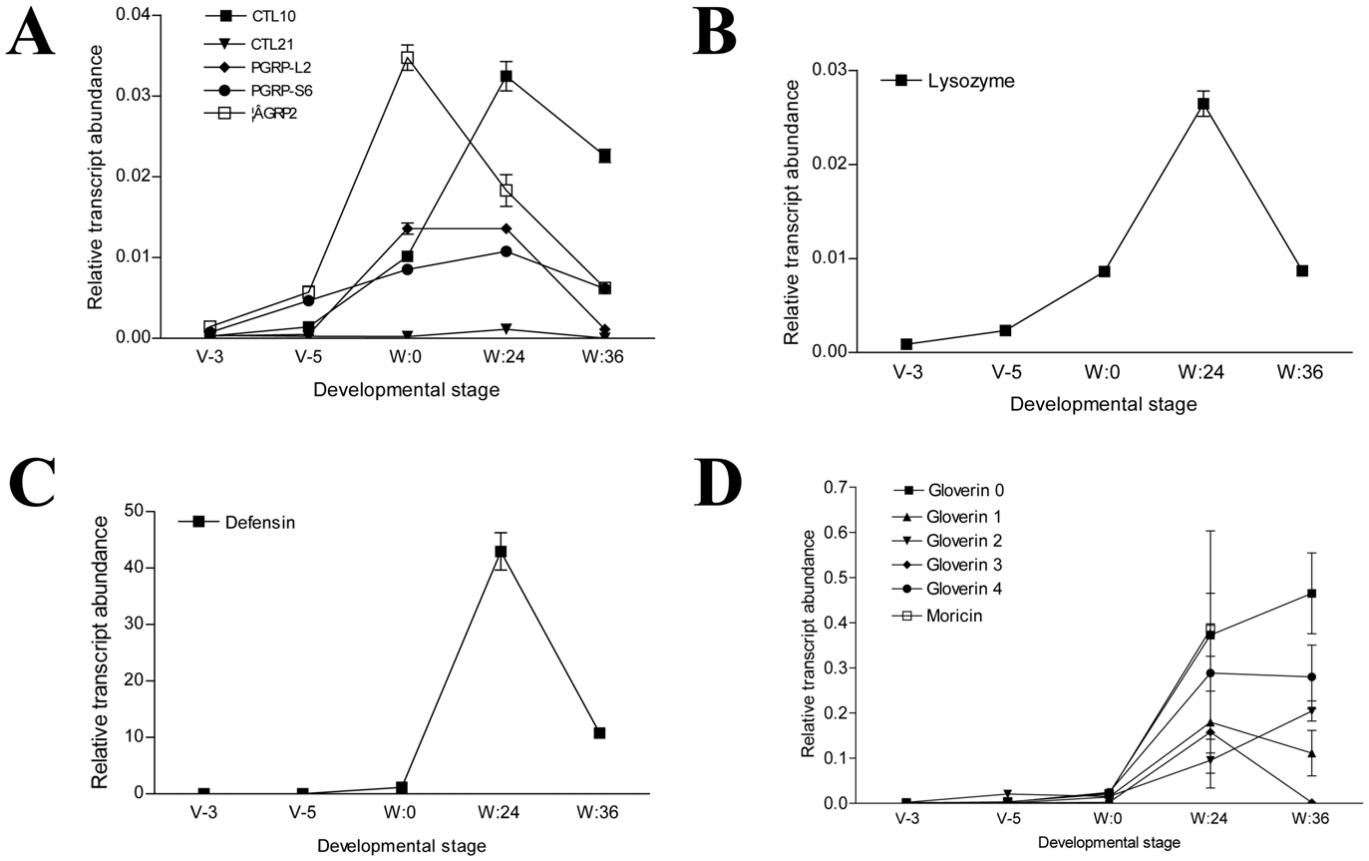
Silkworm midguts produce a cocktail of antimicrobial proteins during the wandering stage. A time-course assay of the transcriptional changes of specific immunity-related genes (A–D). Several antimicrobial peptides and proteins were up-regulated during the wandering stage.

Twenty-five genes related to immunity were differentially regulated in the wandering stage (Fig. 2D). They comprise genes encoding five pattern recognition receptors (PRR), eight antimicrobial peptides (AMP), nine genes belonging to Toll or Imd pathways, and three genes whose protein products probably regulate prophenoloxidase activation. CTL10 and CTL21 that belong to C-type lectins and have humoral and cellular immunity functions [34], [38] were significantly up-regulated in the wandering stage. Before pupation, their transcript levels decreased (Fig. 6A). During the wandering stage, the transcription of the lysozyme gene was up-regulated (Fig. 6B). Lysozyme was also detected in the midgut of the wandering stage but not in the feeding stage (Fig. 1G), which is consistent with the previous report in *M. sexta* midgut [39] and its transcriptional profiling. PGRP-L2 and PGRP-S6 take part in prophenoloxidase activation or antimicrobial peptide production, according to studies in *Drosophila* [40]. Beta-1,3-glucan recognition protein 2 (βGRP2) increased in both mRNA and protein levels (Fig. 6A and Fig. 1G). βGRP can specifically bind to bacterial glycan to trigger the PPO activation pathway [41].

Eight AMP genes were significantly up-regulated during the wandering stage (Table S1 and Fig. 2D). Further analysis of the transcription of these AMPs during development confirmed that these AMPs were significantly up-regulated after the insects entered the wandering stage (Fig. 6B–6D). Defensin increased almost 10,000-fold 24 h after the initiation of the wandering stage (Fig. 6C). Various genes belonging to the Imd pathway as identified in *Drosophila melanogaster* [20] were up-regulated during the wandering stage (Fig. 8A and 8B). However, only a few genes belonging to the Toll pathway were up-regulated (Fig. 8C), and others related to Toll pathway showed no obvious change by microarray or qRT-PCR assays (data not shown). Thus, the Imd pathway may be responsible for producing AMPs in the midgut during the wandering stage.

**Figure 7:**
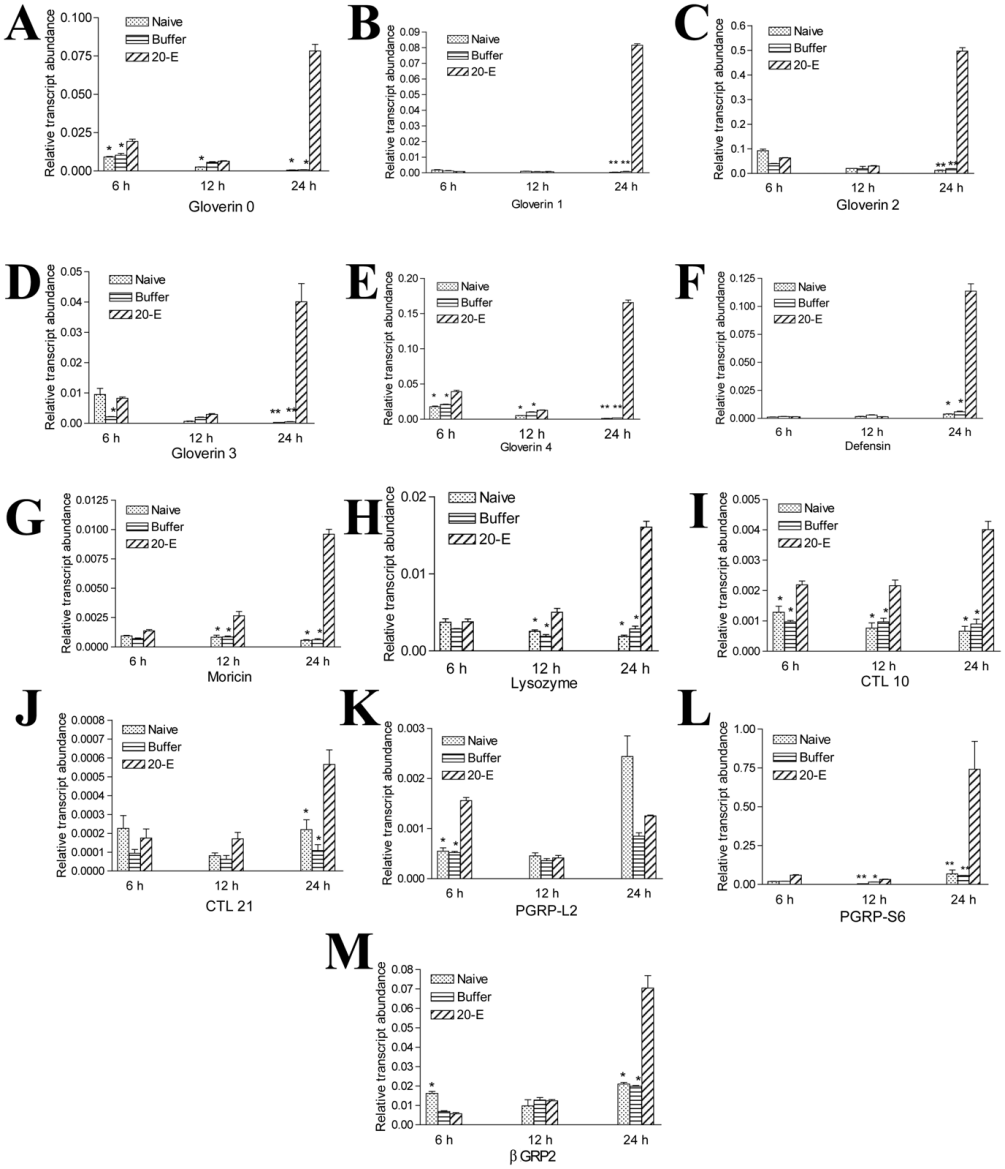
Transcription of immunity-related proteins in the midgut positively respond to 20-E injection. All genes as indicated were significantly up-regulated at different time points after 20-E injection. *p<0.05; **p<0.001.

Hormones also affect the expression of insect innate immunity-related proteins. In *Drosophila*, 20-E controls the transcription of various genes in the fat body [5]. Treatment with 20-E induced the *Drosophila* stable cell line l(2)mbn to express AMP [42]. However, JH and JH homologs, such as methoprene and pyriproxyfen, counteracted the immunity response induced by 20-E in these cells [43]. Therefore, in *Drosophila*, 20-E has a stimulatory effect on AMP production that can be counteracted by JH. In the silkworm fat body, JH has a stimulatory effect, but 20-E has an antagonistic effect, on AMP production [26]. However, in the midgut of the silkworm, the transcription of different AMP genes was up-regulated during the wandering stage (Fig. 6B–6D). 20-E injection significantly induced the transcription of all AMPs at 24 hours (Fig. 7A–7H), which is consistent with the developmental change. In addition, when 20-E was injected into the larvae during the feeding stage, all other genes, pattern recognition receptors (CTL10, CTL21, PGRP-L2, PGRP-S6, βGRP2) were up-regulated (Fig. 7I–7M). The above results indicate that many antimicrobial proteins or peptides are produced when the concentration of 20-E increases in the hemolymph.

**Figure 8:**
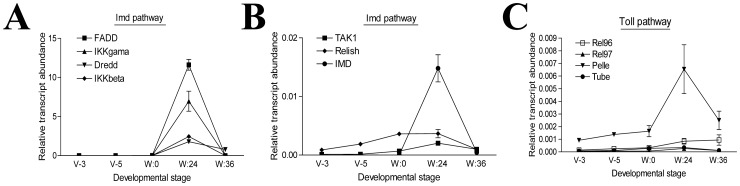
The immune deficiency (Imd) pathway may regulate antimicrobial peptides (AMPs) production in the midgut during the wandering stage. (A, B) All genes of the Imd pathway were up-regulated. (C) A few genes of the Toll pathway were also up-regulated. All others were not changed. Therefore, the Imd pathway might be the main pathway for regulating the production of AMP in the midgut during the wandering stage.

### Midgut Degeneration

**Figure 9:**
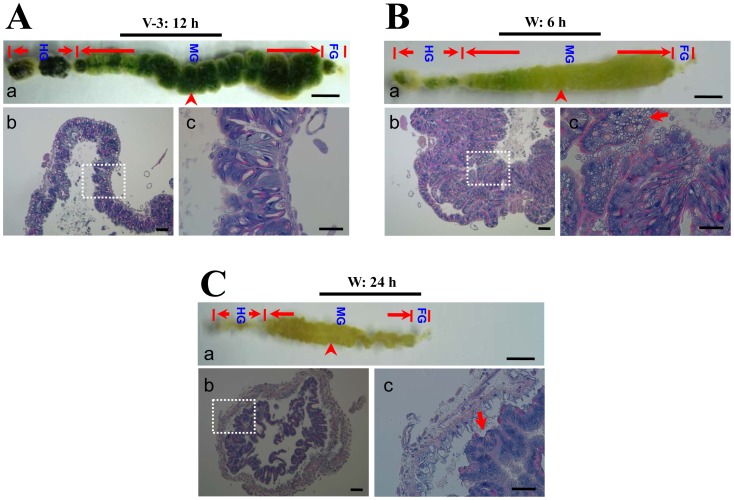
Morphological changes of silkworm midguts during the wandering stage. (A–C) Comparison of the morphology of silkworm guts on the 3rd day of the fifth larval stage (A; V-3: 12 h), and 6 h (B; W: 6 h) and 24 h (C; W: 24 h) after the initiation of the wandering stage. (A–a, B–a and C–a) The whole gut is divided into foregut (FG), midgut (MG), and hindgut (HG). The arrowhead-indicated part of each midgut was sampled for histological study with haematoxylin and eosin which are shown in (b and c) of each panel. Each (b) is a picture with low magnification, and the white-dot-lined area is shown in (c) with high magnification. In (B–c), the arrow indicates a cell full of vesicles probably due to apoptosis. In (C–c), the arrow shows the detached midgut from the basement membrane. Bars: A–a, B–a and C–a: 4 mm; A–b, B–b and C–b: 100 µm; A–c, B–c and C–c: 50 µm.

**Figure 10:**
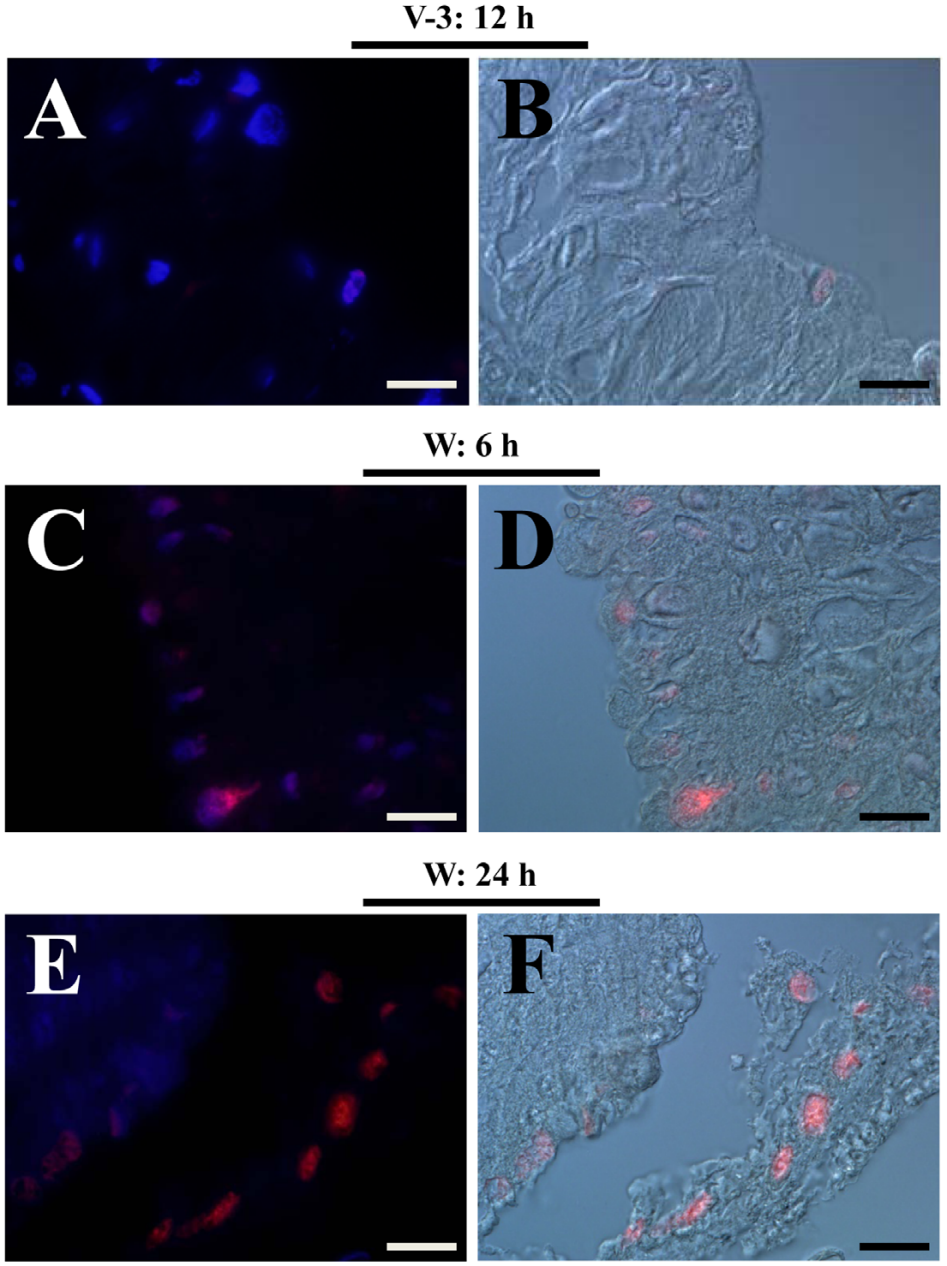
Apoptotic cells in the midgut. Midguts from larvae during the feeding (V-3: 12 h) and wandering (W: 6 h and W: 24 h) stages were sampled. Very few TUNEL-positive (red) cells were found in the midgut during the feeding stage (A, B). However, many cells were undergoing apoptosis in the midgut during the wandering stage (C, D, E, F). Before pupation (W: 24 h), old midguts were observed to slough off from the outer layer of basement membrane. DAPI was used for nuclei counter-staining. All images were merged from pictures taken using red and blue filters or using red and DIC (Nomarski) filters. Bars: 20 µm.

**Figure 11:**
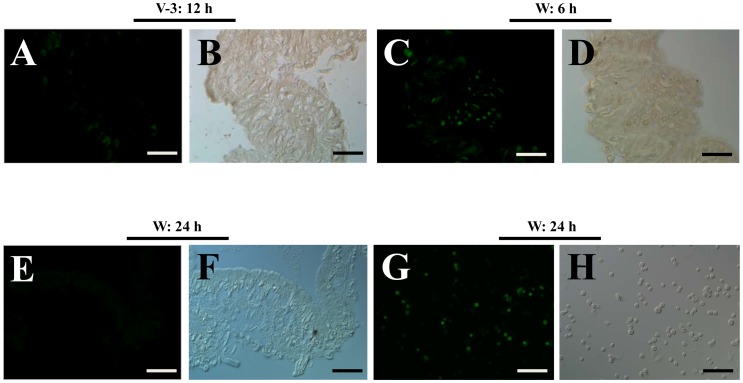
Cell proliferation in the midguts of larvae during the feeding and wandering stages. Green labeling indicates a BrdU-incorporating cell. In the normal feeding stage midgut (V-3: 12 h), very few cells incorporated BrdU (A, B). The midgut at 6 h after initiation of wandering (W: 6 h) had more dividing cells (C, D). At the end of wandering stage (W: 24 h), no cells in the midgut incorporated BrdU (E, F), indicating that cell division there stopped. However, hemocytes from the BrdU injected larvae (W: 24 h) were still stained positively (G, H). Images were taken using a green filter using a fluorescent microscope (A, C, E, G) or under DIC (Nomarski) filter (B, D, F, H). Control experiments performed without primary antibody (anti-BrdU) showed no staining (data not shown). Bars: 50 µm.

The midgut of silkworm moth is under weak condition due to incomplete development if compared with that of a butterfly (Fig. 1A–1F). To date, the morphologies of silkworm midgut during the wandering stage change a lot (Fig. 9). On the feeding stage, the midgut has a normal morphology (Fig. 9A). When entering the wandering stage, the old midgut becomes smaller and is undergoing degeneration (Fig. 9B). The midgut of the feeding stage, as shown in Fig. 9A, is approximately 35 mm in length. But the midgut from an adult, as shown in Fig. 1E, is approximately 4 mm. Therefore, the silkworm midgut is shortened considerably after changing from the feeding stage to the adult stage. Before pupation, the larval midgut is slough off from the outer basement membrane (Fig. 9C). TUNEL staining showed that apoptosis in the midgut started at the beginning of the wandering stage (data not shown) and became intensive at the 6 and 24 h time points (Fig. 9B–9C and Fig. 10C–10F). In the midgut during the feeding stage, there were very few apoptotic cells (Fig. 10A and 10B). In addition to the continuous apoptosis observed in other species of silkworm, autophagy is active during the spinning and pre-pupal stages [15], [16]. However, genes concerned with autophagy were also up-regulated, but the fold changes were lower than the stated threshold for selection (data not shown). Thirdly, when 5′-bromo-2-deoxyuridine (BrdU) was injected to label dividing cells in the midgut, a proportion of cells in the midgut in the feeding stage were positively labeled (Fig. 11A and 11B). We observed that few cells incorporated BrdU after the initiation of the wandering stage (data not shown). The number of positively labeled cells increased at 6 h (Fig. 11C and 11D), but no cells were labeled at 24 h (Fig. 11E and 11F). However, many circulating hemocytes from the same larvae (W: 24 h) had incorporated BrdU (Fig. 11G and 11H). This is probably induced by the decreased capacity of transport because the down-regulation of many transport-related genes happened in the wandering stage (Table S1). It appears that cell apoptosis was continuous in the midgut, but cell division was terminated by the decreased transportation activity of the midgut. However, in the tobacco budworm *H. virescens* (another type of Lepidoptera insect), cell division and cell apoptosis are balanced, and eventually a new fully-functioning epithelium appears during metamorphosis [8]. The midgut of adult butterfly has many layers of cells (Fig. 1C), indicating normal cell replacement during the pupal stage in this species of insect. However, the midgut of the silkworm moth is full of un-excreted yellow bodies that are covered by a layer of cells (Fig. 1F). Therefore, normal cell division to produce a new epithelium with normal absorption and digestion functions would appear to be a prerequisite to stop the degeneration of the midgut during metamorphosis.

The degeneration of the midgut during the wandering stage seems to require certain changes to the transcriptome, as indicated by transcriptional profiling. First, 65 genes concerned with transportation functions changed significantly between feeding and wandering stages (Table S1). Among them, 36 genes are involved in ion transport and 29 are involved in transporting other molecules, such as amino acids, lipids, and sugars. Only six of the ion transport genes were up-regulated; all the others were down-regulated in the wandering stage. This may affect the midgut to have normal functions of ion and other small molecules transport in the wandering stage. Secondly, 19 genes involved in apoptosis showed altered transcript levels between the two developmental stages; 16 were up-regulated during the wandering stage. Growth arrest and DNA damage-inducible gene 45, which is involved in DNA repair, cell cycle control, and apoptosis [44], [45], had an up-regulated level of transcription in the wandering stage. In addition, the transcription levels of eight cuticle proteins also changed (Table S1), among which the levels of seven proteins decreased in the wandering stage (Table S1). Cuticle proteins have been found in the insect midgut, suggesting that this group of proteins may contribute to the growth of the midgut [46].

### Conclusions

The insect midgut is an important organ for food digestion and nutrition absorption. The insect midgut innate immunity has become a research focus because of its heightened immunity against pathogens ingested with foods [21]. During metamorphosis, old tissues are replaced by new ones that might have a different morphology. Surprisingly, the silkworm moth midgut is in a very weak condition (Fig. 1E and 1F). However, many Lepidoptera adults still use their midguts for nectar ingestion and digestion during the adult life stage [8], [17], and the midgut of the butterfly has many layers of cells (Fig. 1C). In addition, some Lepidoptera insects produce antimicrobial proteins in their midgut during the wandering stage by an unknown mechanism [19], [47]. We investigated these aspects by transcript profiling of the midgut during the wandering stage compared with the feeding stage.

Normally, insect hemocytes and fat bodies produce antimicrobial proteins when they are challenged by bacterial components [34], [48]. In *M. sexta*, antimicrobial proteins produced in the wandering midgut kill bacteria according to the *in vitro* assay [18], [19]. The insect midgut contains many bacteria that might induce the production of immunity related proteins during the wandering stages when the midgut is degenerating to give the resident midgut bacteria a chance to contact and challenge the guts. Our qRT-PCR results indicate that genes belonging to the Imd pathway are up-regulated (Table S1; Fig. 8A and 8B). Many genes of the Toll pathway showed no obvious change (data not shown), possibly implying that the Toll pathway is incomplete to work. Thus, the Imd pathway might control AMP production in the midgut during the wandering stage. However, this conclusion still requires further study.

After entering the wandering stage, the expressions of 113 genes associated with various aspects of metabolism and about 59 genes associated with transport of ions and other molecules were down-regulated. The down-regulation of these genes may induce the gradual loss of the normal function of the midgut. Some cells in the early wandering stage midgut incorporated BrdU for a brief period, after which no BrdU incorporation by the midgut cells was observed. On the other hand, the amount of apoptosis increased with time during the wandering stage. Therefore, the down-regulated metabolism and transportation, and the imbalance between cell division and cell apoptosis induce the degeneration of the silkworm midgut in the wandering stage.

## Materials and Methods

### Insect Feeding and Dissection

*B. mori* larvae (Nistari) were reared on mulberry leaves at 25°C under a 12-h photoperiod. Nistari has a period of 36 h of wandering stage (from the initiation of wandering to the time before pupation). The adult butterfly *Pachliopta aristolochiae* (Fabricius) was kindly provided by Dr. Haisheng Yin. The times for different sampling of silkworms are shown in Fig. 2A. According to preliminary work, lysozyme, a very important immunity related protein, was found to be at maximum (protein and transcription levels) in the midgut at 24 h after the initiation of the wandering stage (W: 24 h). However, lysozyme already had a very high level of transcription at the beginning of the wandering stage (W: 0 h) but the protein was not visible until 6 h (W: 6 h) later (see Fig. 1G and Fig. 6B for the above information). In order to cover the genes transcription and protein expression changes as much as possible, we selected larvae at 12 h on day 3 of the fifth larval stage (V-3:12 h), or 3 h after the initiation of the wandering stage (W: 3 h), for dissection for the microarray. The time points for Western blot and qRT-PCR as shown in Fig. 2A were determined according to the preliminary work with lysozyme. To obtain the midgut, the silkworm larvae or moths and butterfly adults were dissected in autoclaved 0.85% NaCl after bleeding. The dissected tissues were washed in fresh 0.85% NaCl three times to remove the hemolymph. The silkworm midguts at the desired ages were dissected in the same way for qRT-PCR, and Western blot assays. Isolated midgut was then pulverized in liquid nitrogen and stored at −80°C in Trizol (Invitrogen, San Diego, USA).

### Oligonucleotide Microarray

RNA isolation, amplification, labeling, hybridization, and microarray imaging and data analysis were performed according to the previously published papers [49], [50]. The microarray, designed by the CapitalBio Corporation (Beijing, China), contains 23,022 probes, each 70 nucleotides (70-mer) in length, corresponding to the approximately 23,000 known and predicted *B. mori* genes [50].

Total RNA was isolated using Trizol reagent according to the manufacturer’s instructions. Total RNA (5 µg) was used to prepare the fluorescent dye–labeled cDNA using cRNA Amplification and Labeling Kit (CapitalBio). The labeled cDNAs were dissolved in 80 µl of hybridization solution (3×SSC, 0.2% SDS, 5×Denhardt’s solution, 25% formamide), and hybridizations were performed in a hybridization chamber (BioMixer ™) overnight at 42°C. Slides were washed two times using washing buffer 1 (0.2% SDS, 2×SSC) and 2 (2×SSC) respectively at 42°C for 5 min. Arrays were scanned with a confocal LuxScan™ scanner and the images obtained were then analyzed using LuxScan™ 3.0 software (CapitalBio). Each experimental group was repeated three times. Data were normalized by the LOWESS method. The filtered data were further examined to find genes that are differentially expressed between samples at two different stages using SAM software [51]. Significance was determined with q-value set at 1%, and ratio of at least 1.5 folds for the signal intensity between experimental sample and control. Gene ontology analysis was performed using Molecular Homological Description System 2.0 (MAS 2.0, http://www.capitalbio.com) [52]. The enzyme-catalyzed reactions were performed using the online pathway relationship database KEGG (http://www.genome.jp/kegg/) [49].

### Immune Challenge

V-3 silkworm larvae were injected with 5×10^6^ formalin-killed *Escherichia coli* cells suspended in sterilized 0.85% NaCl for immune challenge for 12 h [53]. Plasma samples were collected from the larvae to detect different immunity proteins by Western blot as positive controls, as previously described [53].

### 20-Hydroxyecdysone (20-E) Injection

Silkworm larvae on the 3rd day of fifth larval feeding stage (V-3) were injected with 5 µg 20-E (Santa Cruz, CA, USA) per larva [26]. The control larvae were injected with the same volume of solvent buffer. The silkworm larvae injected with 20-E or buffer and naïve larvae were dissected for the midguts as described above for RNA extraction.

### Quantitative RT-PCR (qRT-PCR)

Total RNA was extracted from midguts using Trizol reagent and then treated with RNase-free DNase I. mRNA in 3 µg of total RNA was transcribed into single strand cDNAs using a first strand cDNA synthesis kit (TOYOBO, Osaka, Japan), according to the manufacturer’s protocol. All specific primers were designed using the online Primer3 internet-based interface (http://biotools.umassmed.edu/bioapps/primer3_www.cgi) and are listed in Table S2. qRT-PCR reactions were performed in a 20 µl volume containing 10 µl of 2×SYBR Green Master Mix (TOYOBO), 1 µl of cDNA, 1 µl of each primer (10 µM), and 7 µl of H_2_O. The PCR reaction was performed on a Bio-Rad CF×96™ Real-time System using the following program: 95°C for 3 min, followed by 39 cycles of 95°C for 10 s, 55°C for 30 s, and 72°C for 10 s. Ribosomal protein S7 (rps 7) was used as an internal control. All the samples were measured independently three times. The relative transcription abundances (2^−ΔΔCT^) were calculated according to the equation of 2^−ΔCT^, where ΔCT was calculated as follows: CT target gene-CT rps 7 [15]. GraphPad Prism software was used to produce figures. Columns represent the mean of individual measurements ± SEM (n = 3). Significant differences were calculated with an unpaired *t*-test program by comparing 20-E injection with naïve and buffer injection.

### SDS-PAGE and Western Blot Analysis

Tissues were sonicated in 10 mM Tris-HCl (pH 7.4), and centrifuged at 10,000×g at 4°C for 5 min as previously described [53]. Approximately 10 µg supernatant protein was loaded per lane, and SDS-PAGE and Western blot assay were performed. Antibody against the silkworm lysozyme (a gift from Dr. K. Suzuki; 1∶5,000) [54], or *M. sexta* β-GRP2 (a gift from Dr. M. Kanost; 1∶2,000) [41], or Mouse TAK1 (Santa Cruz, CA, USA; 1∶1,000) was used as the primary antibody, and the AP-conjugated goat anti-rabbit IgG (1∶5,000), or AP-conjugated goat anti-mouse IgG (1∶5,000) was used as the secondary antibody [53].

### *In situ* Apoptosis Detection: the TUNEL Method

Silkworm midguts at different developmental stages were dissected as described above and fixed overnight at 4°C in Bouin’s fluid [53]. Samples were sectioned and deparaffinized as previously described [53]. After deparaffinization and rehydration, the midguts were stained using an In Situ Cell Death Detection kit, TMR Red (Roche, Basel, Switzerland), following the manufacturer’s instructions and as previously described [49]. DAPI was used to counter-stain nuclei. All images were taken using a fluorescent microscope (Olympus BX51, Japan).

### BrdU Labeling and Detection

Silkworm larvae at the desired age were weighed, anesthetized on ice, and then injected with 0.5 mg/g body weight of BrdU (Invitrogen, San Diego, USA), as previously described [55]. The BrdU-injected larvae were sacrificed to obtain their midguts three hours later. Circulating hemocytes were stained to show the positive signal. Midguts labeled with BrdU were fixed and sectioned and stained by a previously-described immuno-staining method [53]. An anti-BrdU (IgG_1_) monoclonal antibody produced in mouse (1∶100; Invitrogen, San Diego, USA) was used as the primary antibody to detect BrdU-labeled cells for 1 h. Rhodamine-conjugated goat anti-mouse IgG_1_ (1∶100; Santa Cruz, CA, USA) was used as the secondary antibody for another 1 h incubation. All images were taken using a fluorescent microscope (Olympus BX51).

### Histological Staining

Insect midguts from different species and at different stages were fixed as described above. Sections (5 µm) were stained with 2% Mayer’s hematoxylin and 1% eosin as described [8].

## Supporting Information

Table S1 **Genes identified by microarray analysis as having ≥1.5-fold higher expression (fold difference) in larvae at 12 h on 3rd day of 5th larval stage (V-3:12 h) than in the larvae at 3 h after the initiation of wandering (W:3 h) stage.**
(XLS)

Table S2 **Primers for qRT-PCR analysis.**
(XLS)
